# Shift of the storm surge season in Europe due to climate variability

**DOI:** 10.1038/s41598-022-12356-5

**Published:** 2022-05-17

**Authors:** Jean-Baptiste Roustan, Lucia Pineau-Guillou, Bertrand Chapron, Nicolas Raillard, Markus Reinert

**Affiliations:** 1 IFREMER, Laboratoire d’Océanographie Physique et Spatiale, UMR 6523 (IFREMER, CNRS, IRD, UBO), IUEM, Brest, France; 2 Direction Générale de l’Armement, Ministère des armées, Paris, France; 3 IFREMER, RDT, Brest, France; 4 Leibniz Institute for Baltic Sea Research Warnemünde, Rostock, Germany

**Keywords:** Ocean sciences, Physical oceanography

## Abstract

Along the European coasts, changes in the timing of the storm surge season are analyzed. Using 10 long-term tide gauges located in western Europe, a consistent spatio-temporal shift emerged in the storm surge season between 1950 and 2000. Temporal shifts are positive (later events) in the North, negative (earlier events) in the South. Extreme surge events occurred about 4 days/decade later in northern Europe, and 5 days/decade earlier in southern Europe. Such a tendency is similar to the one already reported for European river floods between 1960 and 2010. In northern Europe, extreme surges are known to occur during the positive North Atlantic Oscillation phase (NAO+). Identified spatio-temporal shifts likely trace that NAO+ storms tend to occur later between 1950 and 2000. A new index measuring the timing of the NAO+ and NAO− persistent situations is shown to help capture this spatial distribution in the timing of the storm surge seasons.

## Introduction

In the North-East Atlantic, storm surges are fingerprints of extra-tropical storms. As they depend on storm trajectories, storm surge amplitudes greatly depend on the site location. They are larger in northern Europe, with values reaching 3 m in the North Sea, but surges are usually not more than 1 m in southern Europe along the Spanish and Portuguese coasts^1^. The North Sea 1953 flood, with up to 3.3 m measured surge, was one of the most devastating natural disasters in western Europe last century, with more than 2000 reported deaths in the Netherlands and in southeast England^2,3^. More recently, Xynthia storm hit the French coasts in February 2010, generating a major flooding^4^ with 47 deaths and around 10 000 people had to be evacuated^5^. The storm surge reached 1.5 m at La Rochelle harbour, the highest level ever observed since the tide gauge installation in 1997^6^. Combining tide gauge record and historical information, such a value was estimated to occur less than once every 100 years^7^.

The influence of climate change on extra-tropical storms, their frequencies, intensities and trajectories, is thus the subject of a large number of studies. In a warming climate, a possible expansion of the Hadley cell would lead the jet streams and storm tracks to move poleward^8^. Changes in mid-latitudes storm tracks have been reported since the 1980s^9^. Importantly, any change in the frequency, tracks, displacement speed or intensity of storms will then also be traced in the observed storm surge. Many authors thus investigated the variability and trends in storm surges over the last century at quasi-global scale^1,10,11^, at regional scale, e.g. in the English channel^12^, along the European coasts^13^, in southern Europe^14^, along the U.S. coasts^15,16^, in the western North Pacific^17^, or at local scale, e.g. at New York^18^. At first order, changes in storm surges are relative to changes in mean sea level^1,19^. After removing the mean sea level contribution, storm surges display strong interannual to multidecadal variability, and this variability is mainly controlled by the large-scale atmospheric forcing. Indeed, high correlations between storm surges and climate indices are found^1,11,16^. Still, there is no clear long-term (i.e. centennial) trend in storm surges^11–13^, even though trends can be detected on shorter periods. In their review, Feser et al.^20^ concluded that the trends in storm activity critically depend on the analyzed time period. In the North Atlantic, over the last decades, the number of storms tend to increase north of about 55 N (and decrease southward), but considering more than 100 years, there is large decadal variations, and either no trend or a decrease in storm numbers.

While all these studies focus on changes in the intensity and frequency of storm surges, very few studies focus on possible changes in the timing of storm surges. In the North-East Atlantic, storm surges show an expected strong annual cycle. Storm surges more likely occur between December and January^10^. More precisely, at Brest (France), Reinert et al.^21^ reported a clear shift in the storm surge timing. From the analysis of more than 150 years of tide gauge data, extreme events were found to occur three weeks earlier (mid-December instead of beginning of January) in the winter 2000 than in the 1950s. Analysis of additional nearby stations suggests a large-scale process.

The first objective of this paper is to expand on this result and to investigate whether the shift of the storm surge season, revealed at Brest, is also a large-scale spatio-temporal process, distributed along the European coasts. The second objective is to investigate the role of the large-scale climate variability on such a shift.

The paper is organised as follows. The first section describes the ocean and atmospheric data used in our study. The next section describes the two different methods used to compute the timing of the storm surge season, the first one being parametric, the second being non-parametric. Then, we present the results: the large-scale shift observed in the storm surge season between 1950 and 2000, and the link with the North Atlantic climate variability. Finally, we discuss a possible shift in the timing of the storms in the North Atlantic, revealed by the analysis of a new climate persistence index.

## Data

### Sea level data

We used the global sea level dataset GESLA-2 (Global Extreme Sea Level Analysis Version 2)^22^. This dataset, released in 2016, provides high-frequency (i.e. hourly or subhourly) sea level records from tide gauges at as many locations worldwide as possible, i.e. 1355 stations. Data were assembled from 30 providers, the most important being UHSLC (University of Hawaii Sea Level Center)^23^. In GESLA-2 global dataset, the average record length is 29 years, and the maximum length is 167 years in the case of Brest, France.

In addition to sea level records, GESLA-2 provides the surges at each station^1^. These surges, used in our study, are skew surges (and not instantaneous surges), defined as the difference between the maximum observed sea level and the maximum tide prediction during a tidal cycle^24^. The last 15 years were used to compute the tidal constituents, including the annual and semi-annual signals^1^. Note that the tide used to compute the skew surge does not include Mean Sea Level (MSL), so any variation in the MSL, such as MSL rise^25,26^ or interannual variability, remains in the surge data. This concern will be addressed later, adding a linear trend in the parameter formulation (see Eqs 2 and 3).

**Figure 1:**
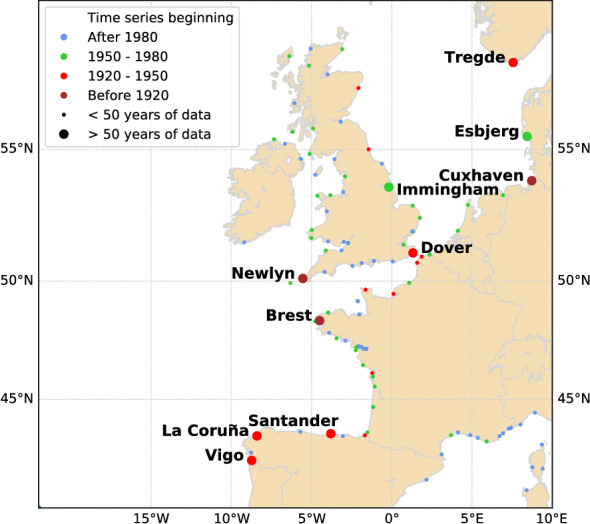
Location of the tide gauge stations from the GESLA-2 database. Labelled stations are the ones used in this study.

We selected stations along the North-East Atlantic coasts with at least 50 years of data, to investigate a possible shift between the 1950s and the year 2000. Note that a year is considered as complete, when it contains more than 300 days of data ($82\%$ complete). This led to selecting 10 stations (Fig. 1). Half of them are located in the North-East Atlantic (Vigo, La Coruña, Santander, Brest and Newlyn), and the other half in the North Sea (Dover, Immingham, Cuxhaven, Esbjerg and Tregde). The time spans for each station are displayed in Table S1 (Supplementary).

### Atmospheric data

To investigate the role of the North Atlantic atmospheric circulation: (1) we used NAO (North Atlantic Oscillation) data, (2) we considered atmospheric data from a weather reanalysis, and (3) we defined and computed a new index, the NAOP (NAO Persistence) timing index.

1. The NAO refers to the main mode of variability of the atmosphere in the North Atlantic^27,28^. This index corresponds to the difference of Sea Level Pressure (SLP) between the Azores high pressure and Iceland low pressure systems, averaged over long periods (e.g. monthly, seasonal, annual). We used the wintertime (December to March) Hurrell station-based NAO Index (retrieved from https://climatedataguide.ucar.edu/climate-data/hurrell-north-atlantic-oscillation-nao-index-station-based, last access: April 2020). This index is computed as the difference of normalized average winter SLP between Lisbon (Portugal) and Stykkishólmur/Reykjavik (Iceland), over the period 1864–2019. In addition to this ‘winter’ NAO index, we computed similarly a ‘daily’ NAO index, based on SLP data from atmospheric reanalysis—see below point (2).
2. We used SLP and 10-meter winds from Twentieth Century Reanalysis version 3 (20CR)^29,30^, a historic weather reconstruction from 1836 to 2015, with a 1° gridded global coverage. The temporal resolution is 3 hours for SLP and winds. We used these data to detect if there is any seasonal shift in the atmospheric data, but also to compute a ‘daily’ NAO index. To do so, we have first checked that the ‘winter’ NAO index computed from 20CR was very close to the Hurrell station-based winter NAO index, over the period 1900–2014. A correlation coefficient of 0.98 between these two indices confirms that 20CR well reproduces NAO climate indices. We then computed a ‘daily’ NAO index similarly to the ‘winter’ NAO index, except that the 20CR SLP data were averaged every day (instead of every winter). This ‘daily’ NAO index will be used further to compute a new index (see below). Note that we used 20CR data only over the 20th century, where we checked that the reanalysis correctly tracks the NAO index.
3. We defined a new index: the NAOP (NAO Persistence) timing index. This NAOP+ (NAOP−) timing index corresponds to the period of the winter, where the persistence in a NAO+ (NAO−) regime is maximum (Fig. 6a and c). It is computed every winter (December–February) as the median date of the longest period with the ‘daily’ NAO over (under) a threshold. Here, the threshold is 2.5 and the daily NAO index is computed from 20CR SLP data (see above). The NAOP+ and NAOP− indices are computed yearly from 1950 to 2000 (Fig. 6b and d). Note that the value of 2.5 for the threshold has been chosen to have at least 3 persistent events per year (an event being considered as persistent when it lasts more than 3 days).

## Methods

The timing of extreme events was computed following two different statistical methods: the first one being parametric (referred as Method 1 in the following), the second being non-parametric (referred as Method 2).

### Generalised extreme value (Method 1)

The first method is exactly the same as the one already applied to Brest, and fully described in Reinert et al.^21,31^ (and also referred as method 1 in their paper). Following previous studies^1,10^ and extreme value theory^32^, a non-stationnary Generalised Extreme Value (GEV) distribution is fitted by Maximum Likelihood on monthly maxima. In brief, considering the monthly maxima values as realizations of a random variable $H_t$, one can expect $H_t$ to follow a GEV distribution: $H_t \sim GEV\big ( \mu (t), \sigma (t), \xi \big )$. The cumulative density function of the GEV is:

1 $$F(h_{t} ;\mu _{t} ,\sigma _{t} ,\xi ) = \exp \left\{ { - \left[ {1 + \xi \left( {\frac{{h_{t} - \mu (t)}}{{\sigma (t)}}} \right)} \right]^{{ - 1/\xi }} } \right\}$$

with $\mu (t)$ the location parameter, $\sigma (t)$ the scale parameter and $\xi$ the shape parameter. Following Reinert et al.^21^, $\xi$ is constant, because this parameter is generally hard to estimate^32^ and does not vary a lot with time^1,11^. However, neither $\mu (t)$ nor $\sigma (t)$ can be considered constant, because their variability is significant. For example at Brest, when fitting the GEV parameters on 20-year running windows (with fixed $\xi$ fitted once on the whole dataset), the variability of $\mu (t)$ and $\sigma (t)$, computed as the ratio of the standard deviation over the average, are both significant: 2.9 and 4.5% for $\mu (t)$ and $\sigma (t)$, respectively.

Similarly to Reinert et al.^21^, we used the following model for the GEV parameters $\mu (t), \sigma (t), \xi$ :

2 $$\begin{aligned} \mu (t)&= \mu _0 + \mu _1 t + \mu _s\cos \big (\omega t + \phi _{\mu }\big ) \end{aligned}$$

3 $$\begin{aligned} \sigma (t)&= \sigma _0 + \sigma _1 t + \sigma _s\cos \big (\omega t + \phi _{\sigma }\big ) \end{aligned}$$

4 $$\begin{aligned} \xi&= \xi _{0} \end{aligned}$$

The parameters $\mu _1, \sigma _1$ test a possible linear trend, especially the one due to MSL rise and mentioned earlier (MSL being part of the GESLA-2 surge data, see the Data section). The parameters $\mu _s, \sigma _s, \phi _\mu , \phi _\sigma$ then help capture the amplitude and phase of the annual cycle, with $\omega = 2\pi .\text {yr}^{-1}$ the annual pulsation. Note that we did not introduce any dependence to the NAO index in the GEV parameters, as done in previous studies^1,10^. The first reason is that the NAO contribution is very small, less than 3% for each parameter at all the stations. The second reason is that it allows to minimize the degree of freedom in our model, and to focus on the annual cycle. As in Reinert et al.^21^, the GEV parameters are estimated with a Maximum Likelihood Estimator (MLE), and we checked that the parameters contribute significantly to the model, with a deviance statistic test at the 5%-level of significance^32^. The Q–Q plots are displayed in the Supplementary Fig. S1.

The annual cycle is represented by the seasonal expectancy $\mathbb {E}_s(t)$:

5 $$\begin{aligned} \mathbb {E}_s(t) = \mu _s\cos (\omega t + \phi _\mu ) + \frac{\sigma _s\cos (\omega t +\phi _\sigma )}{\xi _0}\big (\Gamma (1-\xi _0) - 1\big ) \end{aligned}$$

where $\Gamma (.)$ is the Gamma function. We define the seasonal amplitude as the norm of the expectancy, $||\mathbb {E}_s(t)||_2$, and the timing of the storm surge season as the day corresponding to the maximum of expectancy , $argmax _{t\in [1,365]} \mathbb {E}_s(t)$. This last parameter represents the date of the year when the highest storm surges are expected to occur (i.e. the storm surge season).

To analyse changes in the storm surge season, we fitted our parameters on a 20-year sliding window, with at least 15-years of complete data (i.e. at least 180 months). Thus, for each year, we are able to estimate the timing of the storm surge season. Note that the sliding window size is 20-year, instead of 30-year in Reinert et al.^21^. Sensitivity study show that results are similar with a 30 or 20-year window (see Supplementary Fig. S7a for results with a 30-year window). However, a 20-year window allows to better represent the decadal variability.

### Non parametric statistics (Method 2)

The second method is a simple non parametric method, which does not require any special hypothesis on the data. We consider the 5 highest storm surge events every winter (December to February). The independence criteria between events is of 72 hours. We define the ‘timing of the storm surge season’ as the mean date of these 5 highest events, smoothed with a 20-year running average. Sensitivity study to the sliding window size shows that results are similar with a 30 or 20-year running average (see Supplementary Fig. S7b for results with 30-year running average). An additional sensitivity study was conducted, to test the influence of the number of selected events per year (here 5 events). Results are similar, even if the number of selected events per year vary from 1 to 10 (see Supplementary Fig. S8 for results with 2 and 10 selected events per year).

## Results

### Annual cycle of storm surges

**Figure 2:**
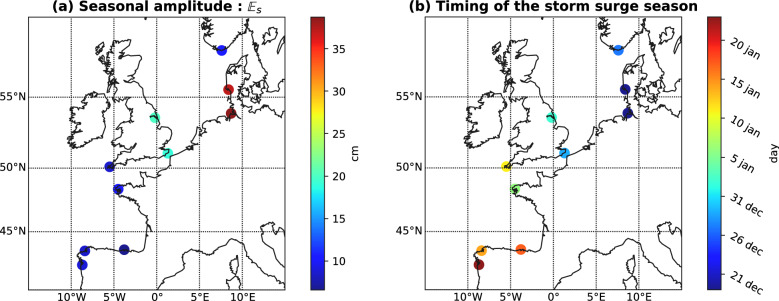
Characterization of the annual cycle of storm surges: (**a**) seasonal amplitude and (**b**) timing of the storm surge season. These two parameters are computed from the GEV-based model (Method 1).

The seasonal amplitude (i.e. amplitude of the annual cycle) and the timing of the storm surge season (i.e. phase of the annual cycle) were estimated at each of the 10 selected stations, fitting a GEV with time-dependent parameters (method 1) on the whole data. The seasonal amplitude varies from 10 to 40 cm (Fig. 2a), with lowest values in the South (Spain). This annual modulation of storm surges is important. It represents roughly 30% of the storm surge signal, whose intensity varies from 30 cm in the South to 80 cm in the North (Supplementary Fig. S2). Concerning the phase of the annual cycle (Fig. 2b), the storm surge season occurs later in the South than in the North (around mid-January in Spain, but rather late December in the North Sea).

### Large-scale shift of the storm surge season

**Figure 3:**
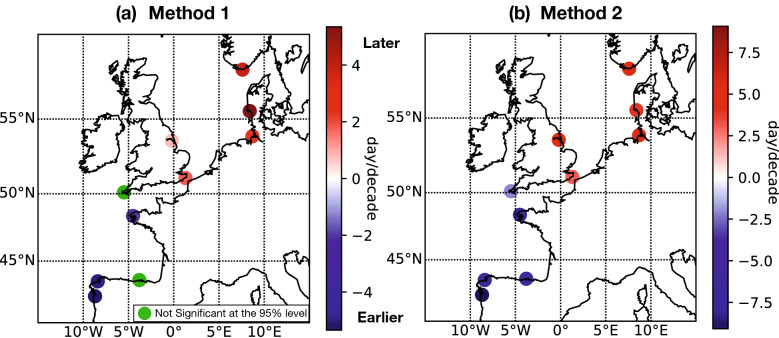
Shift of the timing of the storm surge season between 1950 and 2000, the storm surge timing being computed with (**a**) the GEV analysis referred to as method 1 (**b**) the non parametric method referred to as method 2. The shift corresponds to the linear trend between 1950 and 2000. Note that the color bars have different range.

The timing of the storm surge season was estimated annually, at each of the 10 selected stations, using 2 different methods: a GEV with time-dependent parameters fitted on a 20-year sliding window (method 1), and a non parametric statistic method (method 2, green curve on Fig. 5). The linear trends between 1950 and 2000 were then computed. These trends represent the shift of the timing of the storm surge season, between 1950 and 2000, and are presented in Fig. 3 and displayed in Table 1.

**Table 1:** Linear trends (day/decade) in the timing of the storm surge season at each station, computed with two different methods, the GEV analysis (method 1) and the non parametric statistics (method 2) between 1950 and 2000. Non significant trends are marked with dash. Standard errors are $1\sigma$.

Northern stations						
Stations	Tregde	Cuxhaven	Dover	Esbjerg	Immingham	Mean
Method 1	3.9 ± 0.3	2.3 ± 0.2	1.8 ± 0.3	5.3 ± 0.4	0.8 ± 0.3	2.8
Method 2	4.2 ± 0.4	4.2 ± 0.7	3.1 ± 0.7	3.9 ± 0.6	5.0 ± 0.5	4.1

Southern stations						
Stations	Brest	La Coruña	Newlyn	Santander	Vigo	Mean
Method 1	− 2.0 ± 0.4	− 5.0 ± 0.5	–	–	− 5.1 ± 0.3	− 4
Method 2	− 6.5 ± 0.5	− 7.7 ± 0.5	− 2.0 ± 0.7	− 7.1 ± 0.5	− 9.1 ± 0.6	− 6.5

Results show a large-scale shift between 1950 and 2000, positive in northern Europe (north of 51 N), negative in southern Europe. Importantly, both methods give similar results (Fig. 3a–b). The storm surge season occurs around 4 days/decade later north of 51 N, and around 5 days/decade earlier south of 51 N. At Brest, we found a negative shift of -2 days/decade (with the first method), which is consistent with the shift reported by Reinert et al.^21^, despite the Brest data are here slightly differently processed (we used GESLA-2 data, whereas Reinert et al.^21^ used tide gauge data from French Hydrographic and Oceanographic Service; the raw data (water levels) are the same, but the storm surges slightly differ, due to different processing^1,21^). Note that the values of the shifts are generally stronger with the second method than with the first one: north of 51 N, the shift varies from 3 to 5 days/decade with method 2, but only between 2 and 5 days with method 1 (see Table 1). The same way, south of 51 N, the shift is of around -6 days/decade with method 2, but only -4 days with method 1. Note also that the shift is not significant at two stations (Newlyn and Santander) with method 1, the case of Newlyn being not surprising, as it is located very close to 51 N (i.e. shift close to zero). Importantly, using a different method based on monthly analysis, Reinert et al.^21^ found similar indications for the seasonal shift for stations located around Brest.

In the following, we consider only the timing of the storm surge season computed with Method 2, as the results are similar with Method 1 and 2 (Fig. 3).

### Possible causes

In this section, we show that the timing of the storm surge season is highly correlated with the winter NAO index. Such a correlation suggests a key role of the large-scale North Atlantic atmospheric circulation. In addition, we show that the seasonal shift is already present in the atmospheric data, which has been little discussed until now, to our knowledge.

**Figure 4:**
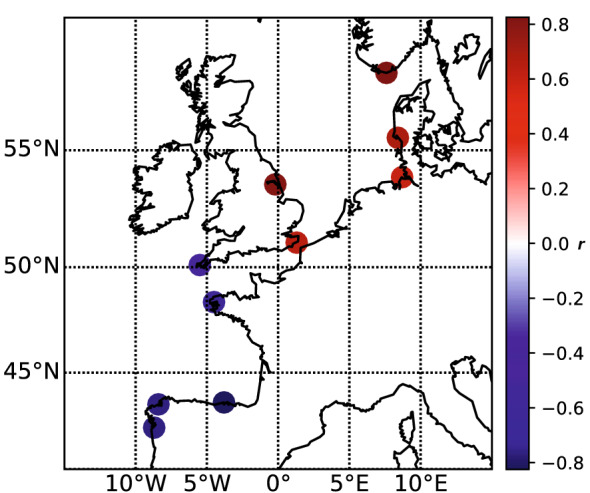
Correlation coefficients, *r*, between the winter NAO and the timing of the storm surge season computed with Method 2 (non-parametric statistics).

**Figure 5:**
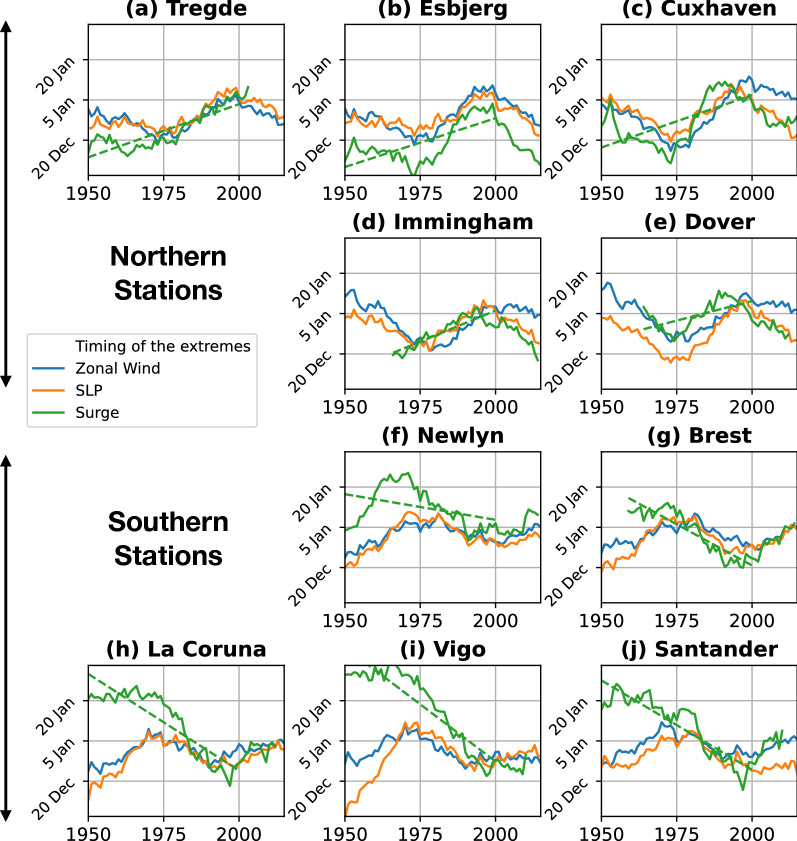
Timing of the extreme zonal wind (blue), extreme low pressure (orange), and storm surge season (green) for each station from 1950 to 2015. The timing is computed with the non-parametric method (method 2). Note that the meteorological data are not extracted at the point closest to the station, but at the point where the correlation with the storm surge is maximum (see text).

At each of the 10 selected stations, we computed the correlations (*r* value) between the timing of the storm surge season (estimated with method 2, see the green curve on Fig. 5a), and the winter NAO index. We did this on the longest period of storm surge data available at each station. Results show strong positive correlation north of 51 N, and strong negative correlation south of 51 N (Fig. 4). On average, the correlation in the North is about 0.75, with a maximum of 0.87 at Tregde. In the South, the mean correlation is -0.71 with a minimum of -0.86 at Santander. These strong correlations suggest a key role of the atmospheric circulation on the storm surge season timing. This is not surprising as storm surges are mainly generated by wind stress and atmospheric pressure. Note that the correlations are similar, whether the timing of the storm surge season is computed with method 1 (Supplementary Fig. S4) or method 2 (Fig. 4).

We then computed the timing of the storm atmospheric events, to investigate if the temporal shift is already present in the atmospheric data between 1950 and 2000. To do so, we analysed wind and SLP time series from 20CR gridded data, exactly the same way we analysed storm surge from GESLA-2 data with method 2 (i.e. the timing of the storm season is computed as the mean date of the 5 strongest winter events, smoothed with a 20-year running average). At each station, the atmospheric time series (wind or SLP) were not taken at the grid point nearest to the station, but at the grid point where the correlation is maximum between the atmospheric data and the storm surges at the station (Supplementary Fig. S3). This allows to take into account that storm surges are not always driven by very local atmospheric conditions, but by remote atmospheric conditions, generating storm surges that propagate up to the coast. In practice, for each station, these correlations are computed between the storm surges at the station and the zonal wind (as well as the SLP) at each point of a 1° × 1° grid, extending from 35 N to 70 N, and 20 W to 20 E, on the period 1980–2000 (Supplementary Fig. S3). The atmospheric times series are extracted from 20CR at the point of maximum correlation, indicated on Fig. S3 (Supplementary) by a green star for the zonal wind, and a green triangle for the SLP. These atmospheric time series are then analysed exactly the same way as storm surges, to estimate the timing of the extreme atmospheric events. Note that we considered only the zonal wind, as we found very small correlations between the surges and the meridional winds (which is not surprising, storm surges being mainly due to the westerlies).

Results show that the timing of the storm surge events (green curve on Fig. 5) is close to the timing of the storm atmospheric events (blue/orange curve for the zonal wind/SLP events on Fig. 5), particularly for the seven stations north of 45 N (located in the English Channel and the North Sea, Fig. 5a–g). For these seven stations, the curves follow well each other, with differences in the timing of the storm surge and atmospheric events lower than 5 days. Only Newlyn shows larger differences, reaching 10 days before the 1980s. Nevertheless, for all of these seven northern stations, the correlation between the timing of the storm surge events and the extreme SLP events is quite high, with significant values larger than 0.5 everywhere (Supplementary Fig. S5b, north of 45 N). The same way, the correlation between storm surge events and extreme wind events is also high (Supplementary Fig. S5a, north of 45 N), except at Immingham and Dover. These two exceptions can be easily explained: along the UK east coast, storm surges are generated by northerly winds behind the storm centre over Scandinavia^33^. For this reason, there is no significant correlation between the storm surges and the zonal wind (Supplementary Fig. S5a). For the three other stations located south of 45 N (along Spanish coasts), the timing of the storm surge events match quite well with the timing of the atmospheric events over the last decades, but not before the 1980s, with large differences exceeding 15 days (Fig. 5h–j). The correlations at these three stations are weak or not significant (Supplementary Fig. S5, south of 45 N). These discrepancies will be explained further, as southern stations may be affected by storms of different weather regimes (see the Discussion).

The good agreement between the timing of the extreme surge season and the storm season suggests that the seasonal shift observed between 1950 and 2000 comes from a shift in the atmospheric data. In the northern stations, this has been confirmed computing the linear trend of the SLP and wind events between 1950 and 2000 (Table 2), exactly the same way than for the storm surge season (Fig. 3b). We found a positive shift of around 2 days/decade north of 51 N. Note that in southern stations, the temporal shift in the meteorological data is mainly positive (Table 2), which is not consistent with the negative shift for the storm surge data (Table 1). These discrepancies are due to the poor agreement in the southern stations, between the timing of the storm surge season and of the atmospheric events (mentioned just above). Note that these discrepancies in the South will be explained further (see the “Discussion”).

**Table 2:** Linear trends (day/decade) in the timing of the storm season at each station for the zonal wind and SLP between 1950 and 2000.

Northern stations						
Stations	Tregde	Cuxhaven	Dover	Esbjerg	Immingham	Mean
Zonal Wind	1.1	1.9 ± 0.05	−	2.2 ± 0.03	−	1.7
SLP	2.6	1.2 ± 0.03	1.3 ± 0.05	2.1 ± 0.01	0.7	1.6

Southern stations						
Stations	Brest	La Coruña	Newlyn	Santander	Vigo	Mean
Zonal Wind	0.6 ± 0.01	0.7 ± 0.02	1.2 ± 0.01	0.14 ± 0.01	− 0.7 ± 0.01	0.3
SLP	1.2 ± 0.03	1.3 ± 0.03	1.7 ± 0.03	0.25 ± 0.02	2.6 ± 0.07	1.4

Trends for the zonal wind are not displayed at Immingham and Dover, as these stations are not impacted by zonal wind^33^ (see text). Standard errors are 1$\sigma$. Note that the meteorological data are not extracted at the point closest to the station, but at the point where the correlation with the storm surge is maximum (see text).

## Discussion

The present analysis reveals high correlations between the timing of the storm surge season and the winter NAO index, these correlations being positive in the North, and negative in the South (Fig. 4). It is well known that a NAO+ regime is associated with stronger than average westerlies in northern Europe, the Atlantic storm activity being shifted northeastward^27,28^. Reversely, a NAO− regime is associated with stronger westerlies over southern Europe. This explains the high positive (negative) correlation between the storm surge intensity and the winter NAO in northern (southern) Europe, found in previous studies^1,10,11^. In other words, storm surges in northern Europe are mostly generated for NAO+ atmospheric situations, whereas storms surges in southern Europe, are mostly generated for NAO− atmospheric situations. To investigate the relation between the timing of the NAO situations and the timing of the storm season, we then consider a new index, the NAOP timing index (NAO Persistence timing index), corresponding to the period of the winter, where the persistence in a NAO+ (NAO−) regime is maximum (see section Data). Linear trends in the NAOP+ (NAOP−) index can then be evaluated between 1950 and 2000. Note that we look at the maximum persistence period rather than the day of the maximum NAO index, to take into account that it is the persistence of the atmosphere in a special state that characterizes a particular weather regime^34–36^.

**Figure 6:**
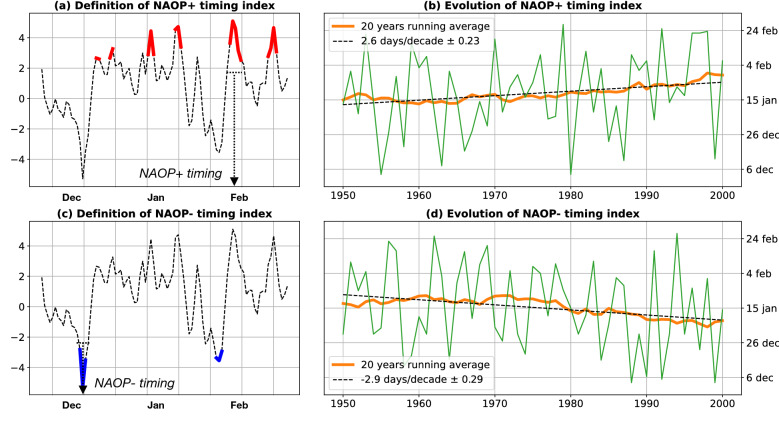
Definition of the (**a**) NAOP+ (**c**) NAOP− timing index. This index corresponds to the median date of the longest period of persistence of the daily NAO+ (NAO−) index over (under) a threshold. Evolution between 1950 and 2000 of (**b**) NAOP+ (**d**) NAOP− index. The linear trend (black dashed line) is 2.6 days/decade for NAOP+ between 1950 and 2000, and − 2.9 days/decade for NAOP−.

Between 1950 and 2000, the NAOP+ timing index presents exactly the same shift than the timing of the storm surge season (Fig. 6b), NAO+ regimes arriving 2.6 days/decade later, against 3 days/decade later for the storm surge season in the North (Fig. 3). The strong NAO+ regimes, which cause larger storm surges in northern Europe, tend to arrive later. Similarly, the strong NAO− regimes, which cause larger storm surges in southern Europe, tend to arrive earlier (− 2.9 days/decade, Fig. 6b). The fact that the NAO+ (NAO−) storms arrive later (earlier), thus explains the seasonal shift of the storm surge season. Note that computing the NAOP index, the sensitivity to the NAO threshold has been checked, for $s \in [1, 3]$: the sign of the shift remains significantly positive for NAOP+ index, only the magnitude is changed (with a maximum difference of one day).

**Figure 7:**
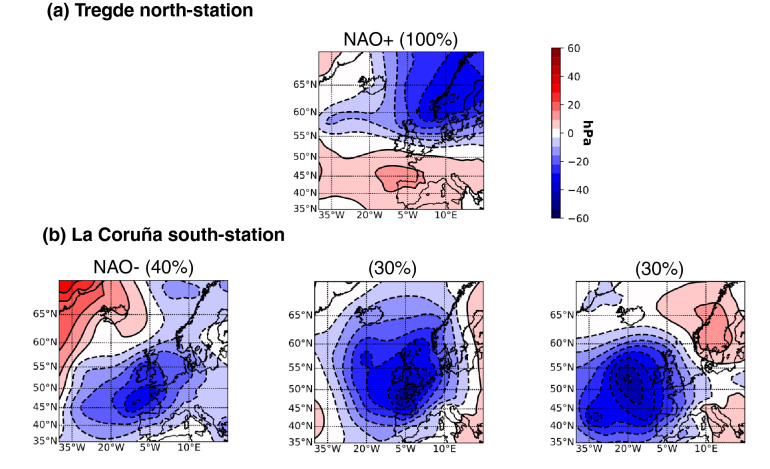
Typical weather situations (SLP anomalies) driving the 15 highest storm surges recorded at (**a**) Tregde and (**b**) La Coruña. SLP anomalies are computed from 20CR, retrieving the average value over the period 1836-2015. See Supplementary Fig. S6 for the corresponding SLP and wind fields.

Our study is mainly based on the correlation with the NAO index. However, North Atlantic atmospheric circulation is more complex than what the single NAO index is able to represent. This complexity explains partly the poor agreement in the southern stations, between the timing of the storm surge season and the atmospheric events (Fig. 5b). Indeed, the NAO represents only the major mode of variability, but 4 climate regimes are generally used to describe the North Atlantic atmospheric variability^28,34,37^. Two of them are the NAO+ and NAO− regimes, the two others correspond to a strong anticyclonic ridge over Scandinavia (“Blocking” regime) and over the North Atlantic (“Atlantic Ridge” regime). For illustration, we computed the typical situations of SLP events at a north representative station (Tregde) and a south one (La Coruña). For both stations, we analysed and classified the SLP situations for the 15 highest storm surge events at the station. In the northern station (Tregde), this led to a single typical situation, close to a NAO+ regime, for all of the 15 events (Fig 7a). Very differently, for the southern station (La Coruña), the analysis led to 3 typical atmospheric situations, generating the largest storm surges (Fig. 7b). The first situation is close to a NAO− regime, whereas the other two situations are close to a “Blocking regime”. However, they are here dissociated, because they represent different storm tracks. Note that there is roughly equal distribution of the 15 events for each typical situation (around 30%). In other words, in the North, the storm surges are mainly due to NAO+ situations, whereas in the South, they are due to a mix of various situations (NAO− and other situations close to a Blocking regime). In the South, the atmospheric time series analysed to compute the timing of atmospheric events (blue and orange curves on Fig. 5b) are impacted by different storms, with different dynamics. This variety of storms may probably partly explain the poor agreement between the timing of storm surge season and of atmospheric extremes in the South (Fig. 5b), and their associated low correlation coefficients (Supplementary Fig. S4, south of 45 N).

## Conclusion

We investigated changes in the timing of the storm surge season along the North Atlantic coasts. We analysed storm surge data at 10 tide gauges, using two different methods. The first one is a GEV analysis (method 1), similar to the one already used at Brest by Reinert et al.^21^. The second one is a non parameteric method (method 2). Both methods give similar results.

Large-scale spatio-temporal shifts emerged between 1950 and 2000 in the storm surge season, positive in northern Europe (north of 51 N), negative in southern Europe (south of 51 N). The storm surge season occurs around 4 days/decade later in the North, and 5 days/decade earlier in the South. Such a tendency is similar to the one already reported for the European river floods, between 1960 and 2010^38^. The floods arrive a few days/decade earlier in northern Europe (in the North Sea), and a few days per decade later in Southern Europe (along the Atlantic coasts).

This work could be extended to additional tide gauges. Indeed, in our study we focused on long-term stations (> 50 years) to investigate the shift over the 1950–2000 period. This led to disregarding many recent stations (see the small points on Fig. 1). Preliminary results on two of them, Milford Haven along the west coast of Great Britain and Malin Head in Northern Ireland, confirm the shift.

Concerning possible causes, the high correlation between the timing of the storm surge season and the NAO first suggests a key role of the large-scale North Atlantic atmospheric circulation. In northern stations, the seasonal shift was already present in the atmospheric data: between 1950 and 2000, the SLP extremes occur later, suggesting later winter storms. The same way, Blöschl et al.^38^ attributed the later river floods in the North Sea to later winter storms. Possibly, storm surge spatio-temporal shifts may trace changes in the storm tracks, accompanying NAO changes, to also likely co-vary with surface temperature and precipitation variability in the North Atlantic sector. Importantly, since the 1990s, the NAO phase is switching from a positive to a negative phase. Consequently, since 2000, the storm surge season tends to now arrive earlier in the North and later in the South. In other words, the observed shift between 1950 and 2000 is not a long-term trend but more likely the signature of large-scale atmospheric decadal variability.

To go further, we investigated the timing of persistent NAO+ and NAO− regimes, through the analysis of a new index, the NAOP Timing Index. We found that this index is also shifting between 1950 and 2000, positively for NAOP+ ($\approx$ 3 days/decade), and negatively for NAOP− ($\approx$ -3 days/decade). For the strong NAO+ regimes, causing larger storm surges in northern Europe, the storms tend to arrive later. Reversely, for strong NAO− regimes, causing larger storm surges in southern Europe, the storms tend to arrive earlier. Note that this new NAOP timing index may be used as a proxy for changes in the storm surge season, but also for other parameters, such as precipitation or flooding.

Finally, we classified the typical atmospheric situations generating storm surges at two stations, located in the North and in the South. Results show that the north-station is clearly characterized by a single typical situation (NAO+), whereas the south-station is characterized by three typical situations (NAO− and two other situations close to a Blocking regime). This variety of storms impacting the south-station explains the seemingly poorer agreement between the timing of the storm surge season and atmospheric extremes in the South of Europe compared to the North of Europe. Further works will have to be conducted to understand more deeply the discrepancies in southern Europe. For example, additional analyses could be conducted on the atmospheric fields, in order to derive indices corresponding to the other situations than NAO, and quantify their influence.

## Supplementary Information

Supplementary Information.

## Data Availability

The GESLA-2 surge dataset^1,22^ analysed during the current study is available on the GESLA-2 website, https://gesla787883612.wordpress.com/gesla2/, the repository link is in the Bibliography section, following the “Marcos, M. and Woodworth, P.L. 2017.” reference, “Supplementary data is here”, https://drive.google.com/open?id=0B4lJ8jUz5qwLOXNBLXFtSmdzLXM (last access: May 2020). The winter NAO index is available in the Climate Analysis Section (NCAR, Boulder, USA) repository, https://climatedataguide.ucar.edu/sites/default/files/nao_station_djfm.txt (last access: April 2020). The Twentieth Century Reanalysis Project version 3 dataset is available in the NOAA repository, ftp://ftp2.psl.noaa.gov/Datasets/20thC_ReanV3/ (last access: January 2021).
