# Supplementary material for: Shift of the storm surge season in Europe due to climate variability
Source: Sci Rep. 2022 May 17;12:8210. doi: 10.1038/s41598-022-12356-5 (PMC9114021; doi:10.1038/s41598-022-12356-5)
Supplement: Supplementary file 1 — Supplementary Information. [file 41598_2022_12356_MOESM1_ESM.pdf]

Supplementary for "Shift of the storm surge season in Europe due to climate variability"

Jean-Baptiste Roustan, Lucia Pineau-Guillou,
Bertrand Chapron, Nicolas Raillard, Markus Reinert

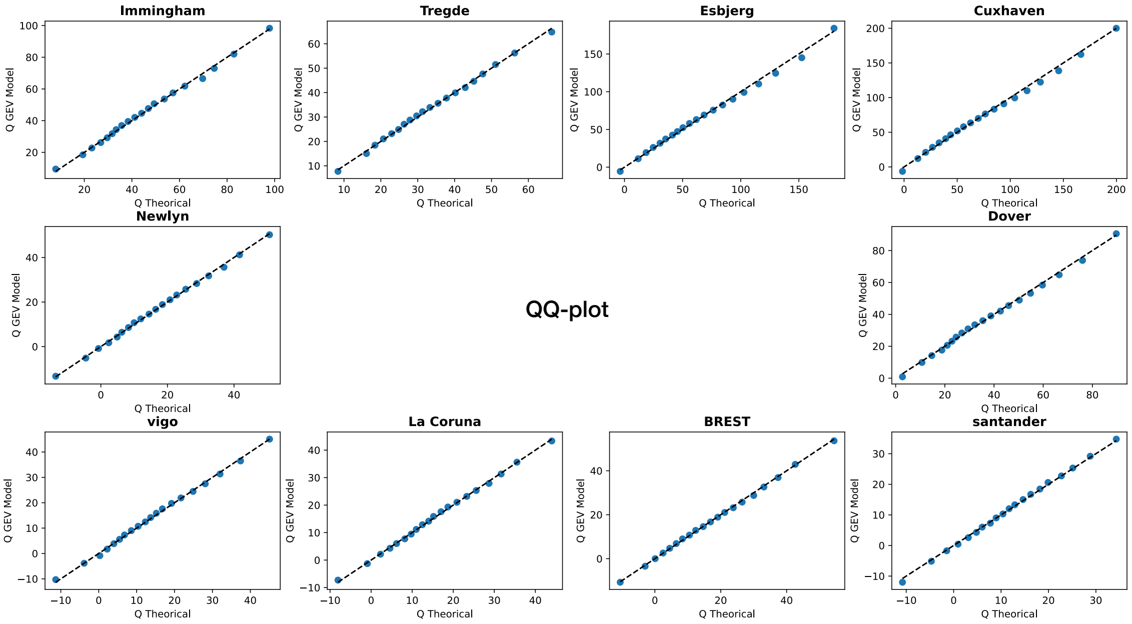

Figure S1: QQ-plots for the GEV model with constant parameters. The fit is made on the whole data set for each station (see Table 1 for the time spans).

Name	Time span
Vigo	1943-2010
La Coruña	1943-2012
Santander	1943-2010
Brest	1846-2014
Newlyn	1915-2014
Dover	1924-2014
Immingham	1953-2014
Cuxhaven	1918-2015
Esbjerg	1950-2015
Tregde	1927-2003

Table 1: Time spans of the tide gauges (from the GESLA-2 database) selected for this study.

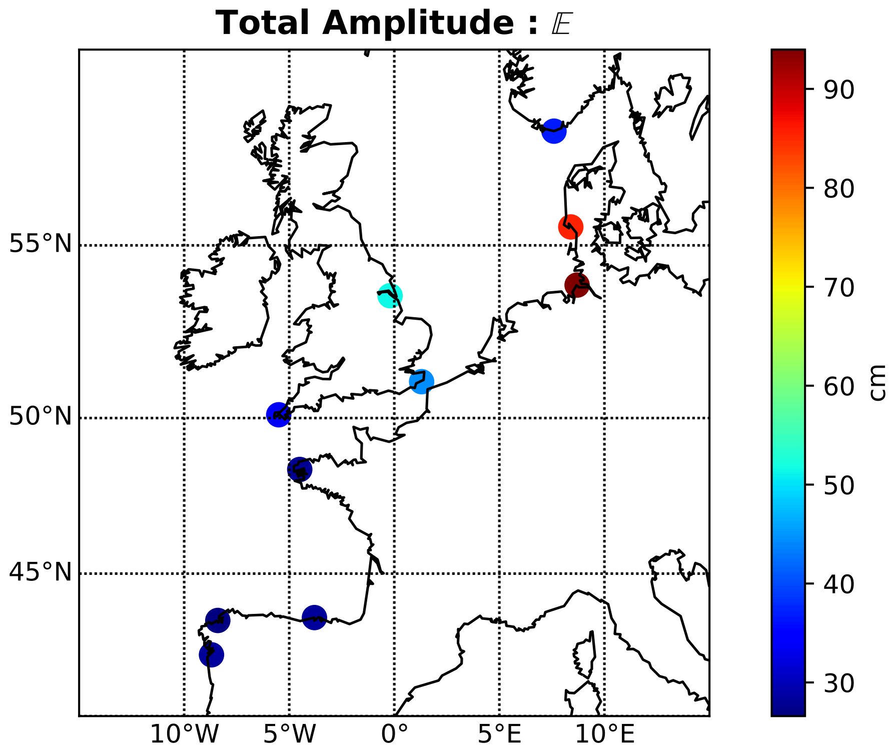

Figure S2: Average of monthly maximum storm surges. This parameter is computed as the expectancy $\mathbb{E}$ of the GEV-based model (Method 1).

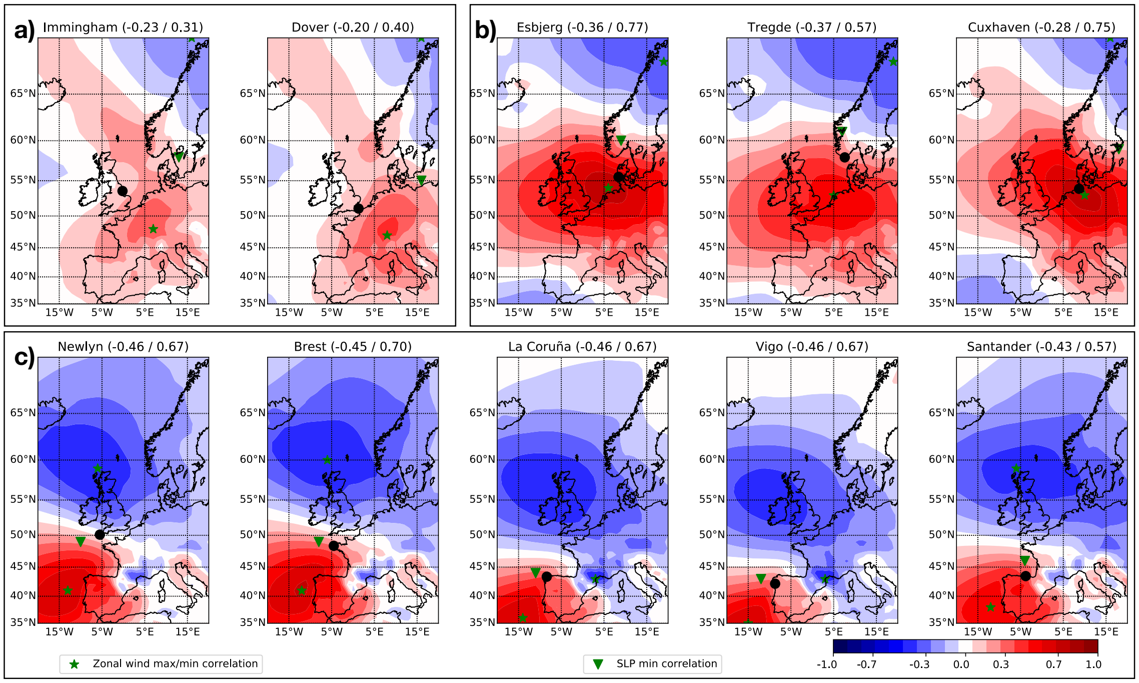

Figure S3: Correlation between the zonal wind and the surge at each station located along the (a) western North Sea (b) eastern North Sea (c) Atlantic coasts. The dark bullet marks the station location. The green star marks the point of maximum correlation between the zonal wind and the surge. The green triangle marks the point of minimum correlation between the SLP and the surge. Note that the distance between the correlation points and the station is always small enough to ensure that the station remains under the storm's influence. The maximum and minimum correlation values for SLP and zonal wind are indicated in the title.

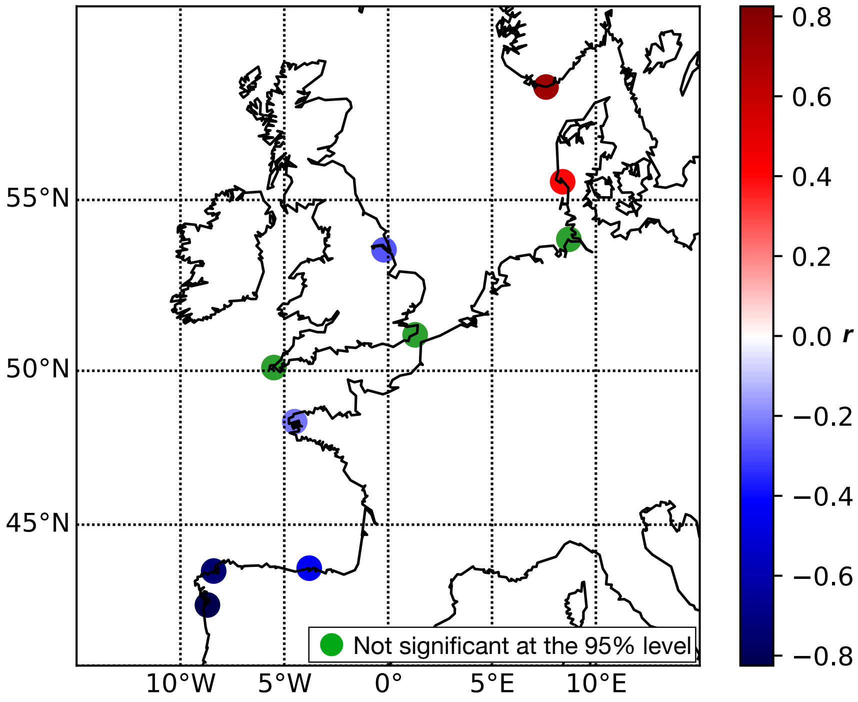

Figure S4: Correlation coefficients, r, between the winter NAO and the timing of the storm surge season computed with Method 1 (GEV).

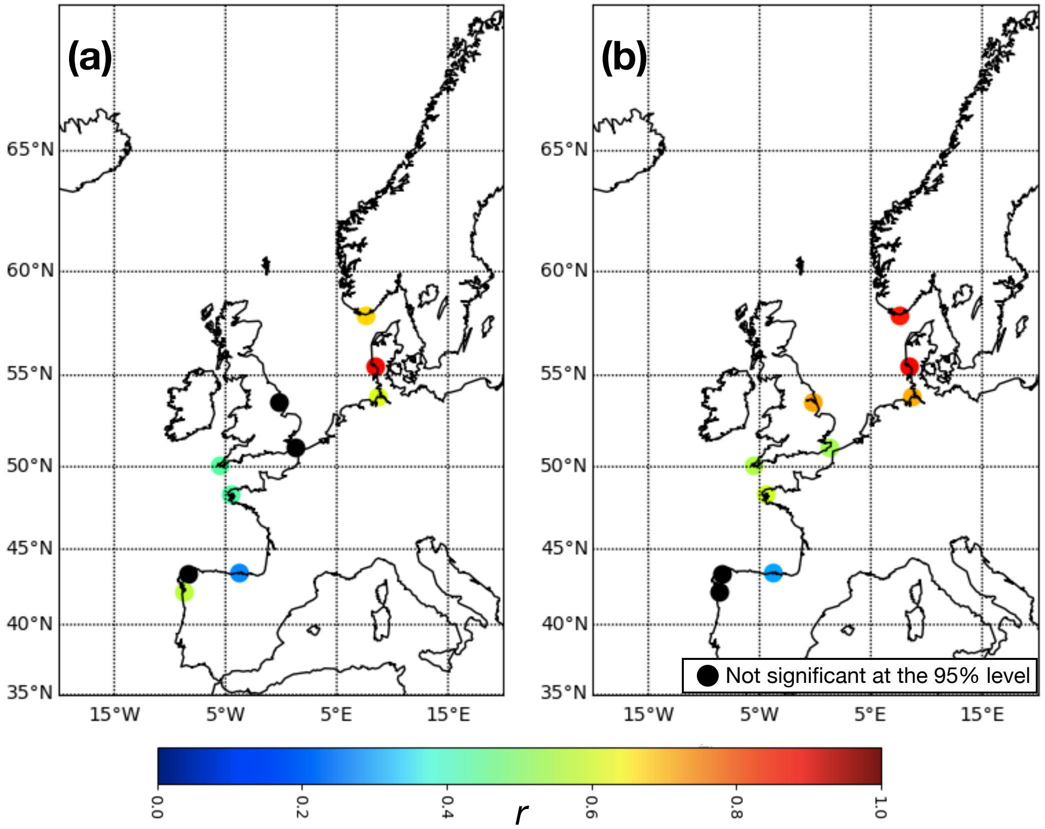

Figure S5: Correlation coefficients between the timing of the storm surge season and the timing of the **(a)** extreme zonal wind season, **(b)** extreme low pressure.

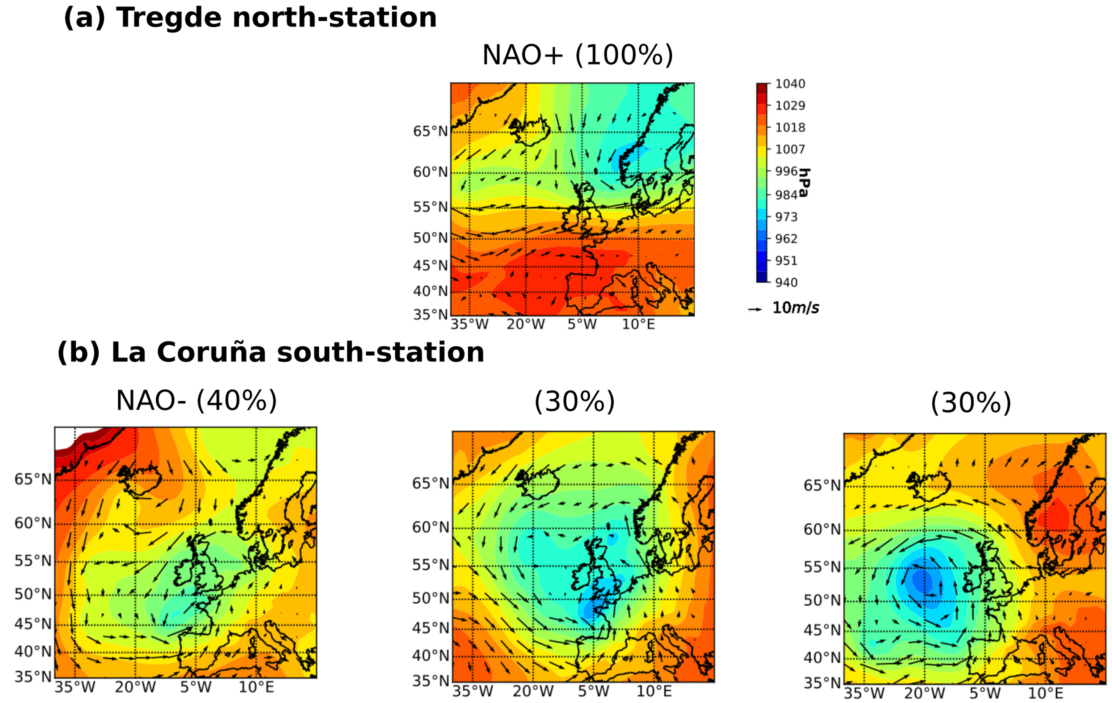

Figure S6: Typical weather situations (SLP and wind fields from 20CR) driving the 15 highest storm surges recorded at **(a)** Tregde and **(b)** La Coruña.

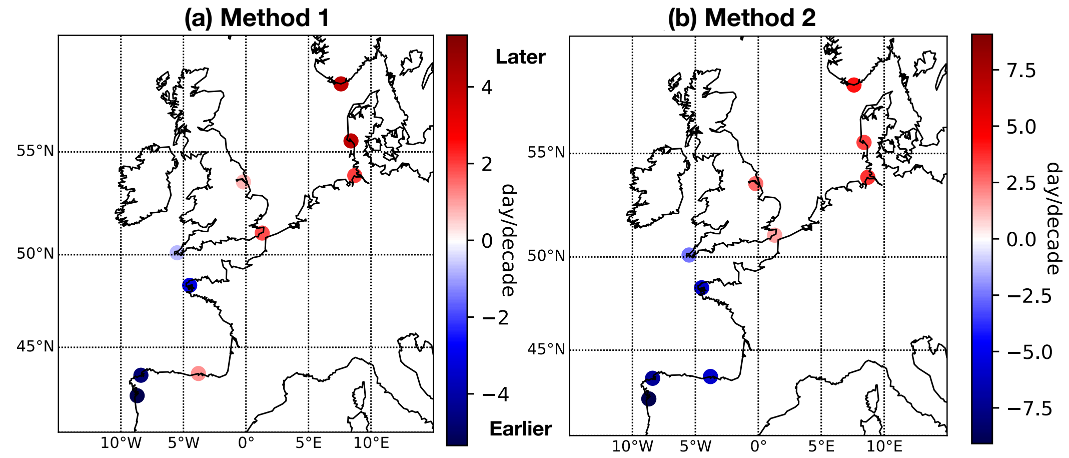

Figure S7: Sensitivity study: same as Fig. 3 of the article, but with a 30-year sliding window instead of 20-year, when computing the timing of the storm surge season.

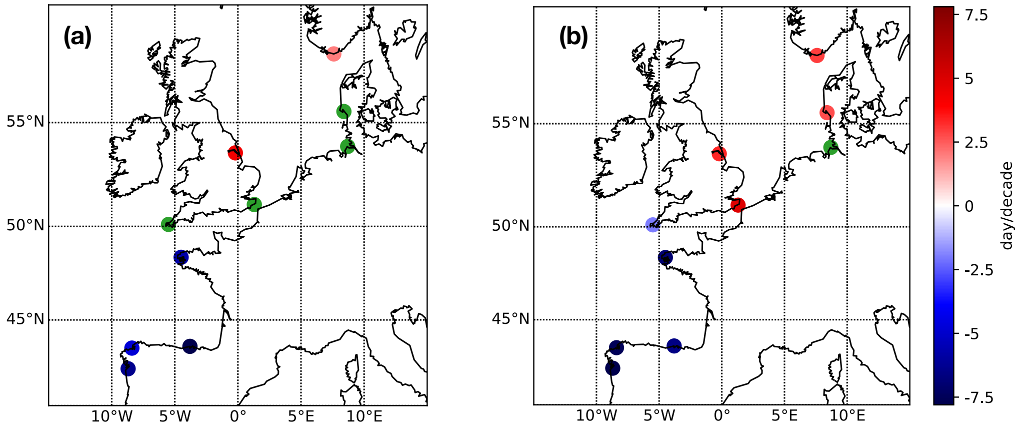

Figure S8: Sensitivity study: same as Fig. 3b of the article, but with (a) 2 (b) 10 selected events per year instead of 5, when computing the timing of the storm surge season.
